# Type-II neural symmetry detection with Lie theory

**DOI:** 10.1038/s41598-025-17098-8

**Published:** 2025-09-29

**Authors:** Alex Gabel, Rick Quax, Efstratios Gavves

**Affiliations:** 1 VIS Lab, University of Amsterdam, Informatics Institute, Amsterdam, Netherlands; 2 Computational Science Lab, University of Amsterdam, Informatics Institute, Amsterdam, Netherlands

**Keywords:** Deep learning, Symmetry detection, Lie theory, Mathematics and computing, Computational science

## Abstract

Understanding symmetries within data is crucial for explainability and enhancing model efficiency in artificial intelligence. This work investigates an approach to neural symmetry detection, specifically leveraging the mathematical framework of Lie theory. Our approach projects data into a low-dimensional latent space, where symmetry transformations can be efficiently applied. By leveraging the matrix exponential, we accurately capture both affine and non-affine transformations, allowing for improved data augmentation and model selection as potential applications. Our method also estimates transformation magnitude distributions, providing deeper insights into the geometric structure of data. Experiments conducted on augmented MNIST demonstrate the effectiveness of our approach in detecting complex symmetries with multiple transformations. This work paves the way for more interpretable and parameter efficient AI models by identifying structural priors that align with the inherent symmetries in data.

## Introduction

Symmetries play a fundamental role in both physics and mathematics, providing deep insights into the laws governing natural phenomena and the structure of mathematical objects. In physics, for example, symmetries underlie conservation laws^1^ and are pivotal in formulating theories such as relativity^2^ and both classical^3^ and quantum mechanics^4,5^. Similarly, in mathematics, symmetries help in understanding geometric structures and solving complex equations^6^. Despite their significance, the integration of symmetry principles into artificial intelligence (AI) remains limited. While certain neural architectures (e.g., convolutional^7^ or group-equivariant neural networks^8,9^) inherently exploit a specific symmetry (translations or a specific group transformation, respectively), a universal approach for detecting and leveraging a broader class of symmetry transformations remains elusive. This is a significant shortcoming, because exploiting symmetries can lead to substantial improvements in model efficiency, interpretability, and performance across various AI applications. In particular, we emphasize its potential use for automatic model selection through structural bias learning in the context of geometric deep learning, what we will refer to as Type-II Deep Learning (cf. Type-II Bayes). In other words, given a neural network $N_\theta$, it could have been engineered^8–11^ or learnt to self-impose^12^ a specific symmetry as a bias through weight-sharing scheme *S*, such that, for set of weights determined by the scheme $\gamma (S)$, its associated neural network $f_{\gamma (S)}$:

1 $$\begin{aligned} N_\theta ({\textbf{x}})\approx \int f_{\gamma (S)}({\textbf{x}}) \, p(S) \, dS, \end{aligned}$$

with a suitable prior distribution over possible schemes and where theoretical aspects regarding an appropriate measure over weight sharing choices are left open to future scrutiny. Practically, a *Type-II Neural Network* can be thought of as an architecture that not only adjusts its weights during training, but has the ability to optimize its weight-sharing scheme for the task at hand.

**Fig. 1:**
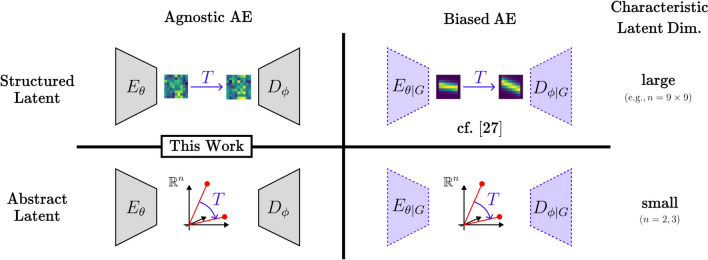
Taxonomy of Lie-Autoencoding Models. Usual approaches exploit the structure of data by using an appropriate neural network for dimensionality reduction. Here, we differentiate between “agnostic” (MLPs) and (geometrically-)biased autoencoders (e.g., CNNs). *G* is used to denote a (geometric) prior, but not it need not necessarily be a *G*-equivariant model. This work investigates models with MLP AEs (left column) while most approaches explored in the literature can be situated in the abstract-biased class (bottom right). Here, *n* denotes the characteristic latent vector size.

In this paper, we tackle deep learning-aided symmetry detection, or *neural symmetry detection*. One can be interested in learning the fundamental geometrical properties of the distribution of data for various applications, ranging from data exploration and topological data analysis^13,14^ to model selection and structural prior learning^15,16^. Motivations include understanding the nature of the data in a broader explainable AI pipeline^17^ (in contrary to processing big data using a black-box) and model efficiency by ensuring a suitable inductive bias, leading to constraints on the hypothesis space while retaining or even improving the expressive power of neural architectures^18^. Traditionally, advancements in deep learning have relied on computational scaling through the accrual of progressively larger data sets^19^. Since this becomes impractical, recent approaches^20^ have opted for reducing the cost of training or the overall size of the model while maintaining competitive performance. In the context of the latter, the irrelevancies of a given task, usually formalized mathematically as symmetry groups^21^, can be exploited by geometric methods that either lead to weight-sharing schemes within the neural network^9,22,23^ or overcompensate with additional computational operations for when the input undergoes the expected transformation^10,24,25^. Modern approaches to symmetry detection focus on learning the most likely symmetry group that relates points in a data set. In previous work^26,27^, the matrix exponential required to quantify the continuous symmetry transformation was approximated in various ways, which sacrifices accuracy for tractability.

We propose instead to evaluate the matrix exponential exactly while avoiding the computationally expensive operation in pixel space by going to a low-dimensional latent space, using a deep autoencoder-like architecture. This offers more control over the behaviour of latent vectors and features by enforcing continuous transformations such as rotations between them, which has been shown to improve the interpretability of features learned by deep models (left column, Fig. 1). Other work that models data geometrically in latent space has interesting connections to neuroscience and information propagation in the brain (top right of Fig. 1, e.g., “topographic” modelling^28^). In this work, in order to learn and control the amount of symmetry bias in our model, geometric information is completely stored in the transformation *T* applied in latent space (e.g., for a Lie group, $T(t)=e^{tG}$), allowing for the application to arbitrarily structured data. This bottleneck in the symmetry detection algorithm removes the need for the engineer to pick a *G*-equivariant neural network for symmetry detection. The transformations are parameterized efficiently, and an extension to non-affine transformations is also possible, leading to more interesting settings such as in the broader class of conformal transformations and more general diffeomorphisms. This has the added benefit of learning suitable representations and has a possible extension to defining the connectivity matrix in the context of structural prior learning (See Section 2.1) and the construction of more general diffeomorphism-equivarianct networks. Splitting the learning of the transformation distribution from the generator is another challenge, and this was possible by introducing a separate network to estimate the transformation magnitudes, creating a seamless end-to-end pipeline. Compared to other works in this field, this model is efficient (low-parameter count for symmetry generator and better than brute force) and interpretable (generators are transferable).

**Fig. 2:**
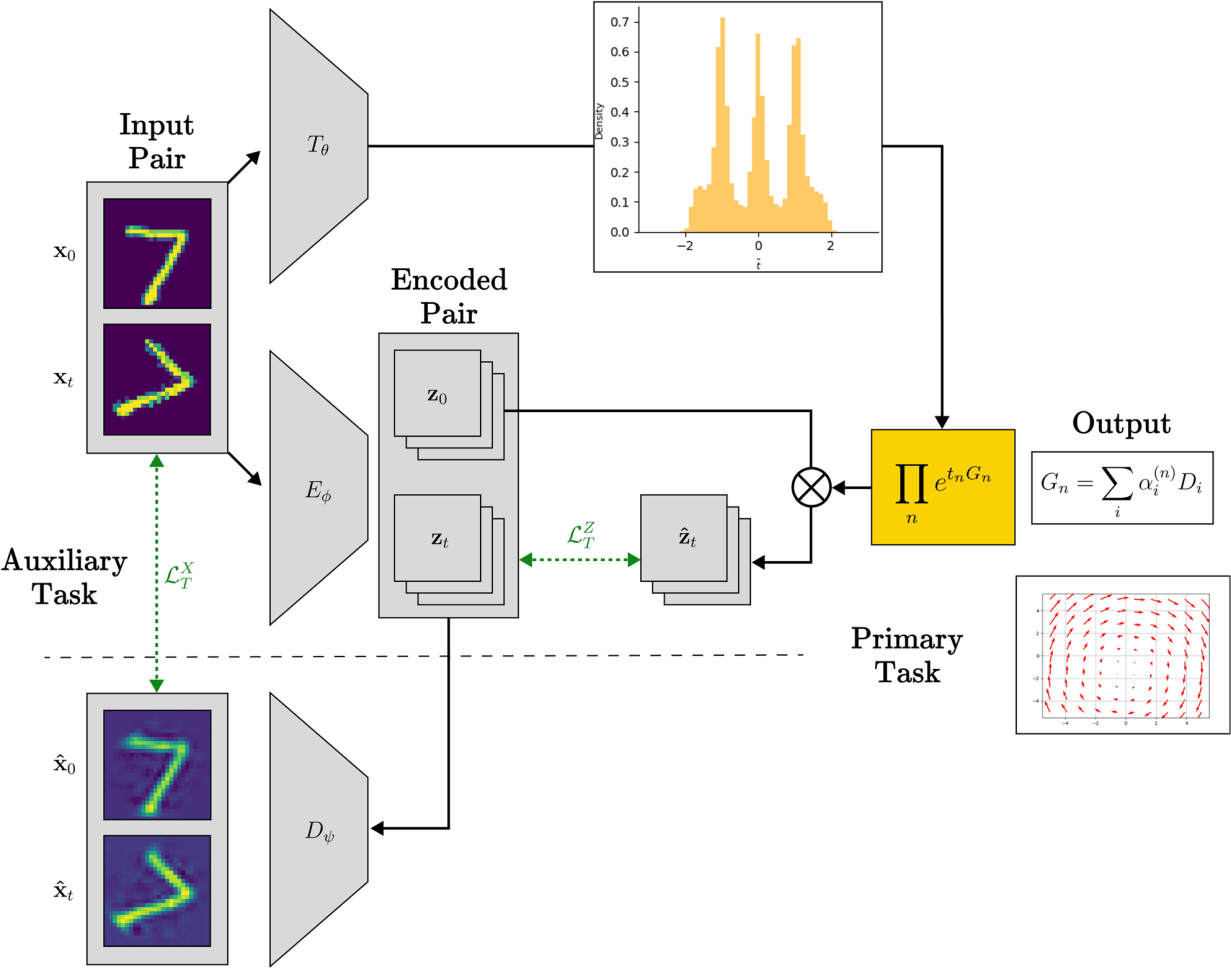
Latent model architecture. The input pair is passed to the *t*-network $T_\theta$ that predicts the magnitude of the transformation (top branch). Simultaneously, the pair is encoded (middle branch) and a consistency loss enforces the latents to be connected by a matrix multiplication with the result of the discretized exponential map. The generator is updated through updating the coefficients $\alpha _i$ related to the chosen basis ${\varvec{D}}_i$. Multiple generators can be applied in series as well, introducing a label *n* for each.

The proposed neural symmetry detector model enforces consistency losses to ensure the encoder-decoder pair remains non-trivial, even when the transformation magnitude *t* is set to zero. At the core of the model, transformations are parameterized using a basis (e.g., affine or quadratic, the latter having the ability to model infinitesimal Special Conformal Transformations), and their magnitudes are estimated by a separate neural network that takes data pairs as input (see Fig. 2). To align transformations in latent and pixel space, we introduce a Taylor expansion-based approximation for matching their estimates, bypassing the computational expense of directly calculating matrix exponentials. This model is evaluated on SyMNIST, a task involving augmented MNIST digit pairs designed to detect symmetry transformations applied during augmentation and estimate the underlying magnitude distribution. We also provide results for SuperSyMNIST, in which the digits in pairs have the same label but are different, including multiple one-parameter transformations and larger canvases. We provide baselines and comparisons with other models, introducing an unsupervised technique ($\alpha$-matching) to link latent space transformations to pixel-space transformations. Experimental results highlight its performance and suggest avenues for further work, including applications to downstream tasks such as Type-II Neural Networks. We also provide all the code for the latent model, which is designed in a modular way to allow for out-of-the-box tweaking and experimenting. Finally, we note that some of these ideas are already reflected in related literature, such as *Noether’s Razor*^29^, latent space symmetry discovery for non-linear symmetry transformations using GANs^30^ or otherwise^31^, attempts at learning weight-sharing schemes using permutation matrices^32^, and the automatic topology design system *AutoML*^33^.

## Method

### Symmetry generators and structural priors

We start with some background knowledge on symmetry detection using the formalism of Lie theory, following an approach by^34^ and recently applied to the same problem in^31^. For the interested reader, for a more thorough introduction to the topic we refer to^35^ and^6^, with a focus on representation theory and differential equations, respectively. Last, we briefly discuss the application of detecting symmetries to structural prior learning for model selection.

#### Lie groups and Lie algebras

The transformations that describe the symmetries are assumed to form *Lie groups*. This means they are sufficiently smooth (*k*-times differentiable, where *k* is usually chosen to be infinity), closed under composition, associative, have a neutral element, and have smooth inverse. These transformations can be defined by the way in which they *act* on objects, namely ${\textbf{H}}:X\times \mathbb {R}\rightarrow X$, with object ${\varvec{x}}\in X \subset \mathbb {R}^n$. In order to apply differential operators to this object, we can treat it as a (canonical) coordinate vector field. Namely, each point is assigned its spatial coordinates as values, and this vector field is clearly $C^\infty (X)$. This also introduces a parameter, $t \in \mathbb {R}$, which is related to the magnitude of the transformation, forming what is usually called a *one-parameter group*. For rotations, this parameter will correspond to the angle, for translations, it will be the distance, etc. Because of continuity in the parameter, we can perform a Taylor expansion of the transformation ${\textbf{H}}$ for small values of *t*:

2 $$\begin{aligned} {\textbf{H}}({\textbf{x}},t)\approx \mathbf {{\varvec{x}}}+t \mathbf {\Gamma }({\varvec{x}}), \quad \mathbf {\Gamma }({\varvec{x}}):= \frac{\partial {\textbf{H}}({\textbf{x}},t)}{\partial t} \biggr |_{t=0}. \end{aligned}$$

We apply the First Fundamental Theorem of Lie^6,34^ in order to make the following claim: $\mathbf {\Gamma }({\varvec{x}})$ defines the transformation and is related to what is known as the *generator* of the transformation. Intuitively, this correspondence between action and generator is due to the constraints imposed on the transformation function being a Lie group. The generator can thus be written as a differential operator as follows:

3 $$\begin{aligned} G = \sum _{i=1}^{n} \Gamma _i({\varvec{x}}) \frac{\partial }{\partial x_i} \end{aligned}$$

That is, if one solves the differential equations $\frac{\partial {\textbf{H}}({\textbf{x}},t)}{\partial t} |_{t=0}$ that characterize the generator, the original transformation function is obtained. More specifically, the solution is the family of functions topologically connected to the identity transformation through continuity in *t*. The generator is an element of the *Lie algebra* of the transformation group and is related to the original transformation by what is called the *exponential map*. This nomenclature emphasizes the connection between the differentiation performed in Equation 2 and exponentiation, easily seen when solving the characteristic equation^6^, i.e.,

4 $$\begin{aligned} \frac{d \mathbf {{\varvec{H}}}({\varvec{x}},t)}{dt}=G{\varvec{H}}({\varvec{x}},t), \quad {\varvec{H}}({\varvec{x}},0)={\varvec{x}}, \end{aligned}$$

as the solution is ${\varvec{H}}({\varvec{x}},t)=e^{tG}{\varvec{x}}$ with $e^{tG}:= \sum _{k=0}^{\infty } \frac{1}{k!} t^kG^k$, where the integer power of *G* is defined by applying the differential operator iteratively. Since we are applying it to a pixelated image or arbitrary signal, we must evaluate *G* in some basis by choosing an interpolation scheme. We discuss this in a later section. The inverse procedure, which extracts the generator from the action as shown in Equation 2, is also referred to as the *logarithmic map*.

We focus on one-parameter groups for two reasons: Ease of implementation and the fact that one such inductive bias is incredibly powerful already. One need not look further than CNNs to conclude that identifying translation as a symmetry of a dataset immediately leads to equivariant models that are superbly successful in practice. Multiple transformations also require additional considerations that relate to the algebra itself, such as closure under commutators^36^, an extension we explored as well for 3-parameter groups.

#### Connectivity and equivariance: a Type-II NN roadmap

   Learning connectivity matrices for deep equivariant models, regardless of whether a symmetry group related to solving the task is given a priori, has been of particular interest to the (geometric) deep learning community^9,10,15,16,32^. It is worth noting that generators can be related to the connectivity matrix, explicitly so for translations, where the shift matrix determines a power series that tiles the weight matrix accordingly. Formally, this involved picking the right representation of the symmetry group of interest, mapping the continuous differential operator formalism described above to linear maps (matrices). We choose to write the partial derivatives in the compact notation, e.g., $\partial _x \equiv \frac{\partial }{\partial x}$.

Practically, for the one-pixel shift matrix *S*, we can write, for $Z\subset {\mathbb {Z}}$:

5 $$\begin{aligned} f_\theta ^L = \sum _{i \in Z} \theta ^L_i S^i. \end{aligned}$$

The above equation defines the weight matrix of one such convolutional layer *L*, with updatable weights $\theta ^L_i$. A collection of multiple power series applied in succession and interlaced with non-linear activation functions *is* the neural network. Schematically, we have6with a total number of layers $\Lambda$, biases ${\varvec{b}}_L$, and activation functions $\sigma$. Note that this finite power series is not able to capture the parametrization of fully-connected layers, as all the elements in the basis need to be related by matrix powers. However, as with the Fourier Transform of the Dirac delta function, it might be necessary to include infinitely many terms in order to correctly converge to an element of the basis of matrices as a vector space, i.e. the null matrix with a single entry equal to one.

In previous work, a main issue was overcoming the computational complexity associated with high pixel count, especially in learning the exponent of a matrix exponential. A second potential issue is introducing strong spatial correlations as a constraint *a priori*, defeating the purpose of learning transformations from scratch. If it is already known that the data is spatially structured, one could introduce continuous coordinates^12^ or use spatial convolutions^10^ immediately, without conveniently ignoring the possibility of spatially unstructured data. This issue is partially alleviated with the matrix exponential method, as learning a generator that corresponds to the zeros matrix leads to the identity matrix, a trivial operation.

### Neural symmetry detection

The biggest technical issue to overcome in the symmetry detection task is learning two separate properties of the data set with a single pipeline: one collective, another pair-dependent. Namely, the generator and the transformation magnitude, respectively. For each dataset, one model is trained and ideally captures the most salient symmetry transformation that relates the data points to each other. The learned symmetry can then be used in downstream tasks. In previous work, especially before the triumph of deep learning techniques, methods such as gradient descent^37^ and expectation-maximization^26^ were used. In this work, we are interested in neural symmetry detection, taking inspiration from works leveraging neural networks as function approximators^27,31^.

#### Defining the task: SyMNIST and GalaxSym

**Fig. 3:**
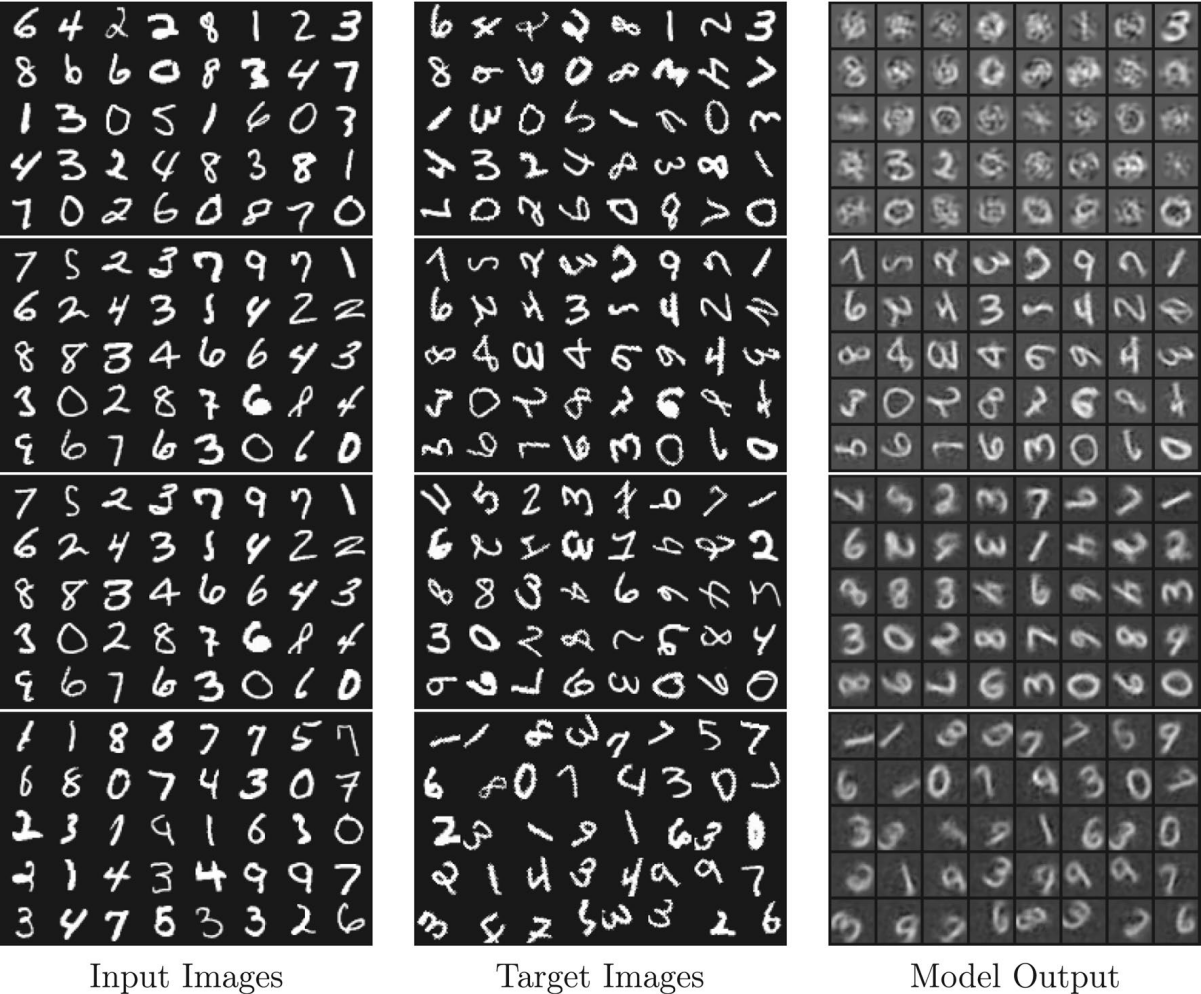
Visual comparison of different models and tasks. The first row shows results from a linear model AE (i.e., single layer MLP), the second row shows a deep AE applied to SyMNIST (augmented sample pairs). The third and fourth rows show SuperSyMNIST, where different digits with the same label are given to the model. The final row illustrates a 3-parameter group of transformations with their corresponding targets and model outputs. The model has learned to capture the overall shape and transformations well.

A symmetry is *a transformation that leaves a certain quantity of interest unchanged*. In order to define the symmetry under consideration, we must state what is being kept “the same”. For the experiments that follow, we consider the classification task and the symmetry that keeps the underlying label identical. The overarching problem, therefore, is learning transformations that map instances of the same class (i.e., data points with the same underlying label) to each other. For the experiments, we introduce the SyMNIST and GalaxSym tasks. The dataset consists of image data, paired with an augmented version of the original image. The classification labels are not used for prediction. The goal is two-fold: (i) extract the type of transformation *G* that was applied, and (ii) estimate the distribution of transformation magnitudes *t* of the seen transformations in the data, which are the “parameters” of the transformation (e.g., rotation angle, scaling factor, etc.). We also have a SuperSyMNIST and SuperGalaxSym extension, in which the second image is still augmented in the usual way but stems from a disparate root image with the same label (See Fig. 3).

**Data availability**:    The datasets analyzed were the widely available MNIST, which can be accessed through many libraries such as torchvision.datasets.MNIST (MNIST can also be found at: https://www.kaggle.com/datasets/hojjatk/mnist-dataset.), and the Galaxy-10 DECaLS dataset (Available at: https://zenodo.org/records/10845026/files/Galaxy10_DECals.h5.). The latter’s images originate from the DESI Legacy Imaging Surveys and were labeled by Galaxy Zoo. The original 128-by-128 RGB images were averaged over the color channels to obtain grayscale version. The torchvision^38,39^ library was used to apply affine transformations to the MNIST images. For non-affine transformations, i.e., the SCT, the torch.nn.functional.grid_sample method was used.

#### Parametrizing the generator

   We can parametrize the generator with any given basis for the functional form of its components, to allow for modelling a broad range of symmetry transformations^40^. In other words, regression is performed on the coefficients of a basis, which can be chosen freely. We wish to have the ability to detect the “typical” symmetries considered in the symmetry detection literature, such as rotation, scaling, and translation, which will be referred to as the *canonical symmetries*, a subset of the affine transformations. This choice, including coefficient sparsity, also places a prior on *p*(*S*) (cf. Eqn. 1), the distribution or hypothesis space of weight sharing schemes *S*. Therefore, we pick a *quadratic basis*, which includes affine transformations, such that the functions $\Gamma _i$ from (3) have the following form:

7 $$\begin{aligned} & \Gamma _x=\alpha ^{(x)}_{c} + \alpha ^{(x)}_{x} \, x + \alpha ^{(x)}_{y} \, y + \alpha ^{(x)}_{xx} \, x^2 + \alpha ^{(x)}_{xy} \, xy +\alpha ^{(x)}_{yy} \, y^2 , \end{aligned}$$

8 $$\begin{aligned} & \Gamma _y=\alpha ^{(y)}_{c} + \alpha ^{(y)}_{x} \, x + \alpha ^{(y)}_{y} \, y + \alpha ^{(y)}_{xx} \, x^2 + \alpha ^{(y)}_{xy} \, xy +\alpha ^{(y)}_{yy} \, y^2, \end{aligned}$$

with learnable coefficients $\alpha _i$. For clarity, in the above we replaced the integers in the subscripts by *c* (for constant terms, related to translations), *x*, and *y* accordingly. The above quadratic basis can capture the canonical symmetries but others (such as shears, compositions, special conformal transformations) as well. Note that one can pick an arbitrarily complicated basis for the expressions given above. This is the major appeal of this approach, and we hope to explore different bases in future work.

**Example 1: Canonical transformations**    In order to encode the three canonical symmetries, one should use coefficients $\alpha$ that yield $G_T=\partial _x$, $G_R=x\partial _y - y\partial _x$, and $G_S=x\partial _x+y\partial _y$, which are the generators of translation (in the *x*-direction), counterclockwise rotation about the origin, and isotropic scaling w.r.t. the origin, respectively. Additionally, one can write down generators for shearing, anisotropic scaling, or combinations of translation and any another of the above transformations.

**Example 2: Special Conformal transformations**    A non-affine transformation that plays an important role in the field of theoretical physics and mathematics is the angle-preserving special conformal transformation (SCT), which has a natural connection to M bius transformations and conformal field theories^41^. The SCT can be thought of as a combination of an inversion w.r.t. the unit circle, followed by a translation by vector $\varvec{b}$, and finally another inversion (we refer the reader to the supplementary material for an example of its effect on a pixelated smiley face). The vector $\varvec{b}$ plays the role of the parameter here, and taking infinitesimally small values leads to the following expression for the generators of the SCT, $G^{(x)}_{SCT}=(x^2-y^2)\partial _x+2xy\partial _y$ and $G^{(y)}_{SCT}=2xy\partial _x+(y^2-x^2)\partial _y$, with the vector pointing in the *x* and *y*-directions, respectively.

#### Interpolation scheme

To apply the operators to a grid, one must write the partial derivatives as matrices. We use the *Shannon-Whittaker interpolation*, as is done in^37^ and^27^. This automatically assumes the function to be interpolated is periodic, although other interpolation schemes could have been chosen. We note that this scheme introduces some aliasing for transformations of low-resolution images, and forms one of the notable limitations of the current model. One could investigate other choices, such as bicubic interpolation, although similar results were obtained. Nevertheless, we pick this interpolation scheme for its ability to perform the transformations of interest using matrix-vector multiplication. Let *I* be some real-valued signal. For a discrete set of *n* points on the real line and $I(i + n) = I(i)$ for all samples *i* from 1 to *n*, the Shannon-Whittaker interpolation reconstructs the signal for all $r\in {\mathbb {R}}$ as

9 $$\begin{aligned} \begin{aligned}&I(r) = \sum _{i=0}^{n-1}I(i)Q(r-i),\\&Q(r) = \frac{1}{n}\left[ 1+2\sum _{p=1}^{n/2-1}\cos \left( \frac{2\pi p r}{n}\right) \right] . \end{aligned} \end{aligned}$$

To obtain numerical expressions (matrices) for $\partial _x$, *Q* can be differentiated with respect to its input. This then describes continuous changes in the one dimensional spatial coordinate at all *n* points, i.e., $[{\varvec{D}}_{\mathbb {R}}]_{ab}=\partial _a Q(a-b)$. The above can be extended to two dimensions by performing the Kronecker product of the result obtained for one dimension with the identity matrix, ${\varvec{D}}_x={\varvec{D}}_{\mathbb {R}} \otimes {\mathbb {I}}$ and ${\varvec{D}}_y={\mathbb {I}} \otimes {\varvec{D}}_{\mathbb {R}}$, mirroring the flattening operation applied to the input images. The parametrized generator for the 2D affine case, for example, looks like:

10 $$\begin{aligned} {\varvec{G}}_\alpha = \sum _{i=1}^6 \alpha _i {\varvec{D}}_i, \end{aligned}$$

where the ${\varvec{D}}_i \in \mathbb {R}^{n^2 \times n^2}$ are the matrices that represent the operators $\partial _x$, $x \partial _x$, $y\partial _x$, $\partial _y$, $x\partial _y$, and $y\partial _y$, respectively. This can easily be extended to arbitrarily dimensional data by adding more factors to the above matrices, as was done above for the quadratic basis. One can see that performing this operation in pixel space scales poorly with signal length (or image width) *n*.

#### Latent model

Learning symmetries using a latent space bottleneck has multiple motivations and has been explored in other research recently^30^. First, learning symmetries from scratch requires learning the relationship between pixels, if one exists, in the data, and this does not scale well with input size as the space of possible connectivities is factorial in nature (cf. the permutation group). Second, and related to the previous point, is the poor scaling of the size of the generator that needs to be matrix exponentiated in order to preserve the continuous nature of the symmetry transformations. Finally, the encoder should ideally learn to remove unimportant information from the data, such as background information in an image classification setting. We opt for an adapted autoencoder architecture, whose latent space we use to learn the transformations.

In our model, the autoencoder design allows for the model to keep relevant information in the latent space and transform this according to a transformation, a result of the exponential map, it shares with all other input pairs (Fig. 2). The model takes an image as input and reconstructs the transformed image as output. In order to get an estimate for the parameter (e.g. rotation angle) a separate network is trained together with the autoencoder. The parameter estimate is then passed to the matrix exponential function that is then used to matrix multiply the latent patch(es). Finally, the latent patch(es) are decoded, and a reconstruction loss is enforced on the output-transformed image pair. The learnable parts of the model can be grouped as follows:

1. The encoder $$E_\phi$$
2. The decoder $$D_\psi$$
3. The *t*-network $$T_\theta$$
4. The parametrized generator ${\varvec{G}}_\alpha = \sum \alpha _i {\varvec{D}}_i$

The loss function consists of a part that ensures both the *n*-dimensional pixel-space and *d*-dimensional latent-space vectorized data pairs transform to each other. For images ${\varvec{x}}_0, {\varvec{x}}_t \in \mathbb {R}^{n^2}$ and ${\varvec{G}}_\alpha \in \mathbb {R}^{d^2 \times d^2}$, one can write these as:

11 $$\begin{aligned} {\mathcal {L}}^X_T({\varvec{x}}_0, {\varvec{x}}_t)&=\Vert g_\psi \circ e^{T_\theta ({\varvec{x}}_0,{\varvec{x}}_t){\varvec{G}}_\alpha } \circ f_\phi ({\varvec{x}}_0) - {\varvec{x}}_t\Vert ^2, \end{aligned}$$

12 $$\begin{aligned} {\mathcal {L}}^Z_T({\varvec{x}}_0, {\varvec{x}}_t)&=\Vert e^{T_\theta ({\varvec{x}}_0,{\varvec{x}}_t){\varvec{G}}_\alpha } \circ f_\phi ({\varvec{x}}_0) - f_\phi ({\varvec{x}}_t)\Vert ^2 \end{aligned}$$

and can also be written as ${\mathcal {L}}^X_T({\varvec{x}}_0, {\varvec{x}}_t)=\Vert \hat{{\varvec{x}}}_t - {\varvec{x}}_t\Vert ^2$ and ${\mathcal {L}}^Z_T({\varvec{x}}_0, {\varvec{x}}_t)=\Vert e^{t{\varvec{G}}_\alpha } {\varvec{z}}_0 - {\varvec{z}}_t\Vert ^2$. The model allows for various numbers of latent patches (cf. channels) to be transformed in parallel in the latent space.

$\varvec{\alpha }$-**matching**:    We also include a loss term that enforces the generator in the latent space to be close to the one in pixel space is introduced. Since calculating the matrix exponential in pixel space does not scale well w.r.t. data size, a different approach needs to be used. Hence, this $\alpha$-*matching* term uses the learned $\alpha _i$ from the latent space (Eq. 7) and places them in a generator for the pixel-space, using a Taylor expansion, to compare its effect on the vectorized input, formally

13 $$\begin{aligned} {\mathcal {L}}_\alpha =\left\| \left[ {\mathbb {I}}+t{\varvec{G}}'_\alpha +\frac{1}{2}t^2({\varvec{G}}'_\alpha )^2+ \ldots \right] {\varvec{x}}_0 - {\varvec{x}}_t\right\| ^2, \end{aligned}$$

where the prime denotes the basis ${\varvec{D}}'_i$ evaluated in pixel-space, i.e. ${\varvec{G}}'_\alpha , {\varvec{D}}'_i \in \mathbb {R}^{n^2 \times n^2}$. If one wishes to enforce sparsity on the coefficients of the terms in the generator, a sparsity loss (LASSO) can be added in order to enforce correct behavior in the symbolic regression. This assumption helps with understandability and interpretability of coefficients, but is also a prior on *S*.

Furthermore, we include the standard reconstruction loss term for each of the inputs in the data pair individually, ${\mathcal {L}}_R({\varvec{x}})=\Vert D_\psi \circ E_\phi ({\varvec{x}})-{\varvec{x}}\Vert ^2$, to train the autoencoder. This loss term simply encourages the encoder to learn to reconstruct individual image inputs well, as usually done with autoencoders. Thus, this term ignores the exponential on the latent space. The total loss, therefore, is:

14 $$\begin{aligned} {\mathcal {L}}({\varvec{x}}_0, {\varvec{x}}_t) = {\mathcal {L}}^X_T({\varvec{x}}_0, {\varvec{x}}_t) + \lambda _Z{\mathcal {L}}^Z_T({\varvec{x}}_0, {\varvec{x}}_t) + \lambda _{R}\big [{\mathcal {L}}_R({\varvec{x}}_0) + {\mathcal {L}}_R({\varvec{x}}_t)\big ] + \lambda _\alpha {\mathcal {L}}_\alpha + \lambda _{L} ||\varvec{\alpha }||_1. \end{aligned}$$

In order to avoid the ambiguity in the exponent of the matrix exponential (namely, $t{\varvec{G}}=st \times {\varvec{G}}/s, \forall s \in {\mathbb {R}}_0$), the generator is normalized by enforcing the coefficient vector to have unit norm during training. I.e., $||\varvec{\alpha }||^2=1$, where $\varvec{\alpha }$ is the vector made up of the coefficients $\alpha _i$. A consequence of this choice is that the *t*-network is expected to produce zero-valued outputs for image pairs that are not path connected.

**Multiple transformations**:    In order to allow for more complicated transformations that do not form one-parameter groups, a straightforward extension to the proposed model can be accomplished by adding more factors of matrix exponentials. This allows for transformations connected to the identity in a continuous way, charting out the space of possible transformations defined by the parameters associated with the various generators. If one wishes to enforce the Lie algebra structure, one can include, by design, multiplication by a closure factor: $e^{t_{ij} [G_i,G_j]}$. This implementation is present in the codebase. Since no conclusive results were obtained from experiments with this setup, and for the sake of clarity of the presentation of the results, we leave this for future work. We note that reconstructions were still very good, so further research in this direction should prove fruitful. Another option would be to enforce this algebraic constraint in the loss.

#### Computational complexity

The computational complexity of the latent Lie symmetry detector model depends on the choice of loss formulation. With the first order Taylor term enforcing $\alpha$-matching, we have a matrix exponential of $O(L^3)$ compared to $O(N^3)$ for brute force, where $L$ is the size of the latent patch and $N$ is the total number of pixels or features in each data point. In the latent model, there are now 3 additional NNs which scale predominantly with input size for this setting. Depending on the tapering of the MLP, the complexity is always better than $O(N^2)$. Activating the second order Taylor term in the loss introduces an $O(N^3)$ scaling for the matrix-matrix multiplication. Either this term can be omitted or more efficient procedures for calculating this term can be implemented in future work. Nevertheless, the computational burden is still lower than that of calculating a matrix exponential. (We refer the interested reader to the comparisons in supplemental material.)

## Results

### Implementation and the SyMNIST/GalaxSym tasks

Here, the implementation of the latent model is quickly sketched. For further implementation details, we refer the reader to the Appendix (A). We note that code will be made available with extensive comments and documentation. Transformations are applied using the affine function from the torchvision library^38^ and torch’s grid_sample for more non-affine transformations. The images can also be placed on a larger canvas, which is particularly useful when translations are part of the applied augmentations. The magnitude of the transformation is the parameter sampled from the chosen distribution. This procedure allows for flexibility in testing a neural symmetry detector, as the distribution can be arbitrary and, in theory, so can the transformations. In these experiments, we will focus on detecting combinations of various affine transformations and a non-affine transformation, the SCT. We focus on applying our model to the SyMNIST task, but we note the latent model works equally well with Galaxy-10 DECaLS data. Samples for both SyMNIST and SuperSyMNIST are shown in Fig. 3.

The proposed model offers several advantages over previous approaches. Unlike earlier works that rely on supervised learning for estimating symmetries^27^, or are restricted to small image patches^26^, our method enables unsupervised detection of both affine and non-affine transformations directly from images. Additionally, the proposed framework can handle arbitrary and multi-modal distributions of transformation magnitudes and multiple one-parameter group transformations, which significantly broadens its applicability compared to methods that assume uniform or low-modal (typically 2 or 3) transformation magnitude distributions^42,43^. Furthermore, the model directly evaluates the learned symmetries rather than relying on downstream tasks for validation^15,32^. By learning both the underlying generators and the distributions of transformation magnitudes, our approach provides a comprehensive solution for symmetry detection that is robust, transferable across datasets, and capable of handling complex transformations. Finally, we experimented with abstract (2-dimensional) latents and did not observe as high a quality of reconstructions as for the structured (patchified) latents, most likely due to the low dimension of the latent space. This issue could potentially be fixed in future work by separating the shape (2-dimensional) from the residuals (finer details). (A figure showcasing the 2-dimensional latents is provided in the supplemental materials.)

### Transformation magnitude distribution

**Fig. 4:**
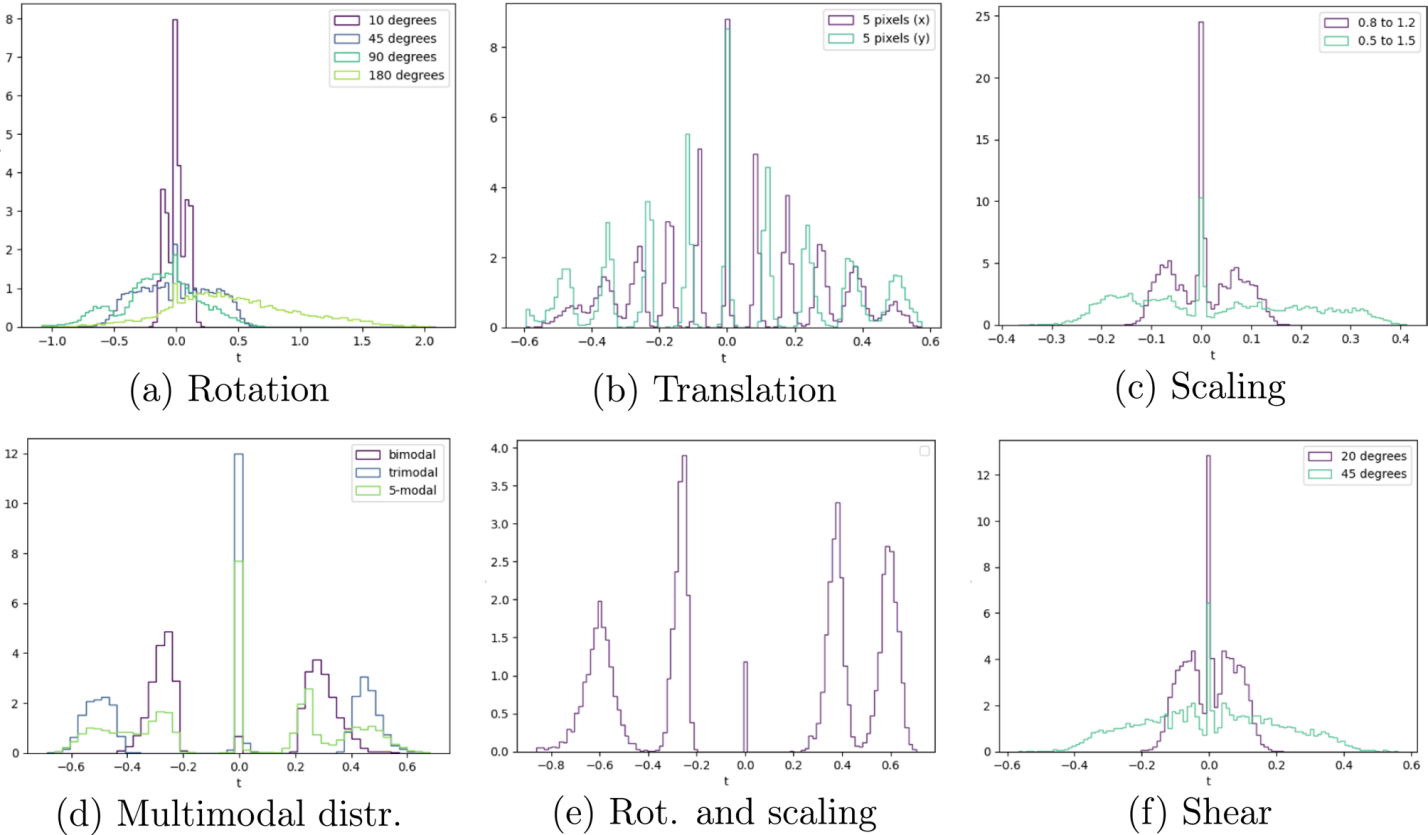
Learnt transformation magnitude histogram for various transformations. Magnitudes for more complicated distributions, compositions, and non-canonical transformations were also learnt. Results for (d) and (e) were produced by categorical, the others, by uniform sampling of *t*.

Histograms of learned transformation magnitudes reveal correct modes for same-sample pairs (Fig. 4), but performance degrades for dissimilar pairs, i.e., SuperSyMNIST. This is most likely due to the fact that MNIST digits are all differently aligned, are not perfectly centered, and that the MSE loss does not capture semantic similarity in pixel space very well, especially for small transformation magnitudes. It seems like the distributions capture aliasing artifacts in the small angle and translation setting (Fig. 4b). Note the broadening of the range (in radians) of the learned distribution when sampling angles in the rotation setting, which breaks down for larger values (Fig. 4a). Peaks are also visible at the origin, even when no pair relates to such an identity transformation (as in Fig. 4d and e). We qualitatively see good shape reconstruction for uniform distributions for small angles and scaling transformations (Fig. 4a and c). Clearly, such a qualitative assessment is not rigorous enough for a thorough analysis.

**Fig. 5:**
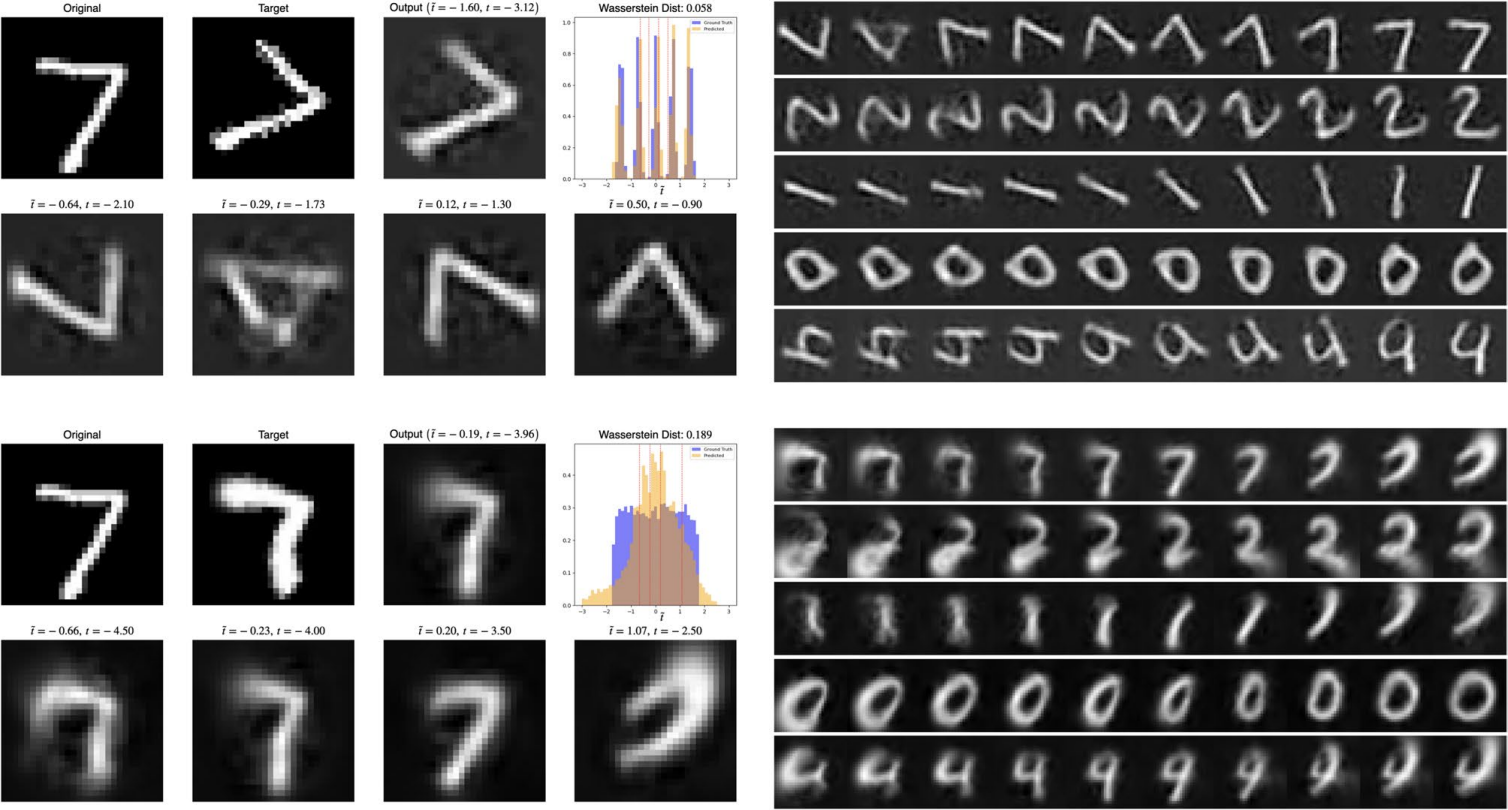
Top row: 5-modal SyMNIST with rotation. (Left) Image-target pair and output of the model used for the auxiliary task, including the sampling versus the recovered distribution of normalized transformation parameters $\tilde{t}$ (above). A traversal through *t*-space and reconstructions for unseen transformations, corresponding to red dotted lines in the *t*-distribution plot (below). (Right) Fine-grained variation of *t* emphasizes topological anomalies, with transitions being less problematic for digits such as “1”, “2”, and “0” but more apparent for symmetric digits like “7”. Plots are for equally spaced $t \in [-2, 0]$. Bottom row: (Left) Visual comparison of SuperSyMNIST for uniform SCT distributions (non-affine model, ${\textbf{a}} \in {\mathbb {R}}^{12}$), highlighting versatility but sensitivity to sharp distribution edges. (Right) Continuous variation of *t* for $[-5, -2.5]$.

The Wasserstein distance for the normalized distributions provides a quantitative metric for learned distributions. By looking at test-time samples, we observe interesting behaviour when traversing in *t*-space (Fig. 5). Certain transitions between modes are seemingly continuous is pixel-space, but anomalous can be found in certain models. For a 5-modal distribution, the model seems to learn intrinsic symmetries in the digits as a way to rotate them (top). We also show a drawback of the model, namely that it is quite tough to learn exact distributions, in particular for uniform distributions in the SuperSyMNIST case (bottom). In the latter, this is probably due to the MSE loss not being the most suitable objective for this task. On average, however, the model seems to recover the correct transformation and the reconstructions at test time look accurate, especially considering the overall shape.

**Fig. 6:**
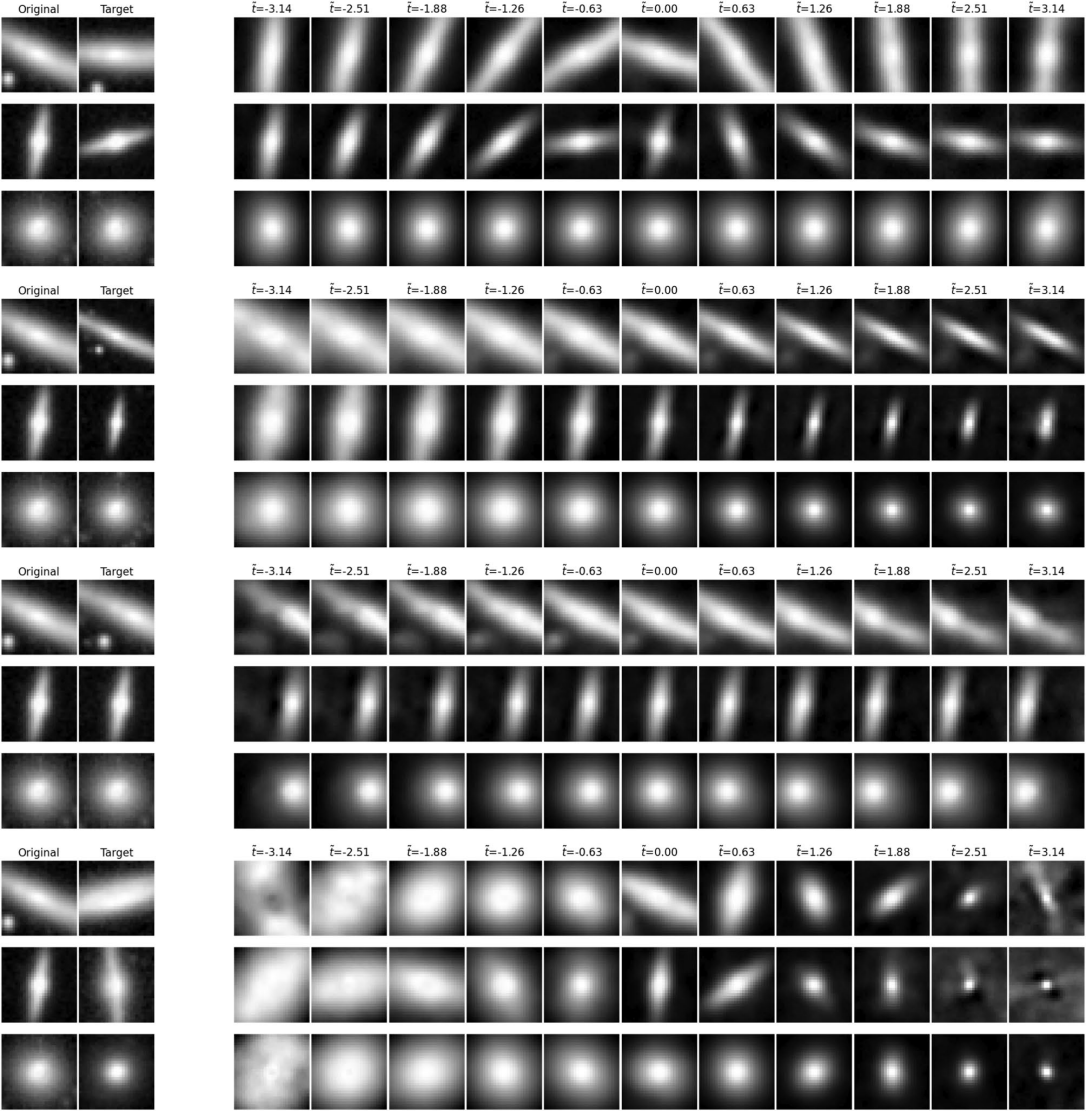
Visualization of learnt transformations on three GalaxSym and one SuperGalaxSym tasks: rotation, scale, shift, and super-rotation (similar to no augmentation) respectively. The model clearly learns to reproduce the transformation well in pixel space. In the SuperGalaxSym case, it seems to learn scaling as well as rotation, reflecting the distribution of sizes of galaxies in a given class.

### Symmetry detection performance

**Fig. 7:**
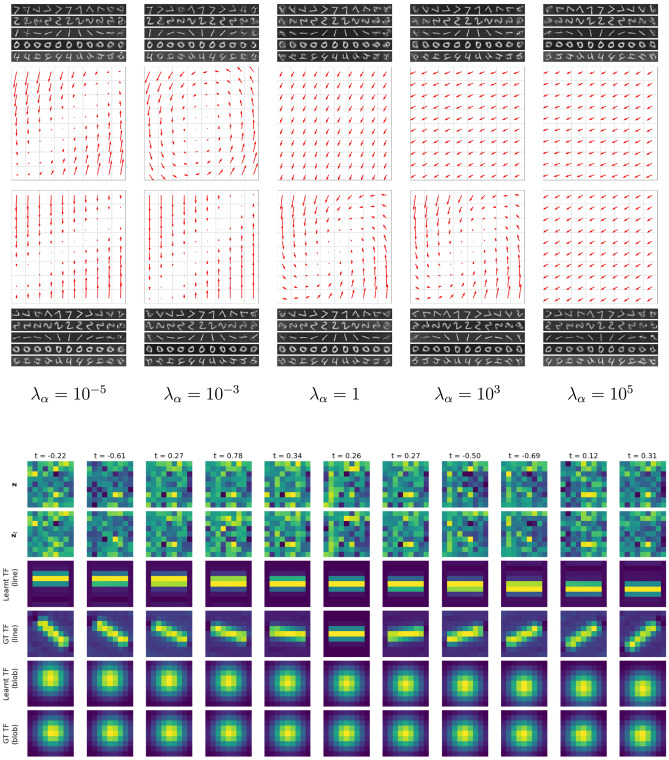
(Top) Comparison of reconstructed images (first and fourth rows, for *t*-values regularly spaced between $-\pi$ to $\pi$) for a model trained on rotations with $t \sim {\mathcal {N}}(\mu =0^{\circ }, \sigma =45^{\circ })$ and generator flow fields visualizations (second and third rows) for different values of $\lambda _\alpha$ values using order 1 (first two rows) and order 2 (last two rows) Taylor approximations. (Bottom) When omitted, or when $\lambda _\alpha$ is too small (here: $\lambda _\alpha =10^{-3}$), alpha matching cannot tie the transformation in pixel space to those in latent space, possibly resulting in a mismatch, such as a shift when a rotation was desired. (Actual latents and predicted *t*-values, including the action of the exponential map on line segments and Gaussian blobs is shown for an order 2 model trained on rotated GalaxSym pairs.)

Visually, the transformations at test time look good and similar to the ground truth. In most failure cases, the location and overall shape is still correct, but the more detailed elements are either missing, blurry, or in an incorrect location. By plotting the vector fields associated with the learnt generator, we can visualize the flow of the transformation (Fig. 7, top). In order to compare to the ground truth generator, we split the coefficients up in drift, diffusion, and non-affine terms. This makes it easier to identify a correct transformation, such as a rotation, that has drifted due to a spurious non-zero translation coefficient. Additionally, we can look at the latent patches the model learnt and evaluate the transformations on a sample patch (Fig. 7, bottom). In cases where a rotation was not recovered, we do see evidence of different solutions to the symmetry detection task: for certain flow fields, and low transformation magnitudes, one can mimic the correct transformation, e.g., by pushing and pulling the sides of the image using a shear rather than a rotation (Fig. 7, bottom left). From looking at the latents, it is also not obvious whether the model has learnt any geometrically structured signals. Qualitatively, in GalaxSym, we note the pixel level transformations look exceptional both within and just outside the *t*-distribution (Fig. 6). Generators are the closest to the ground truth for translation and scaling, but rarely correct for rotation (checked by flagging imaginary eigenvalues, signalling a center of rotation, in the diffusion matrix). Perhaps the alpha matching term requires more tuning. The LASSO loss term only seemed to have a marginal effect regarding performance, hampering reconstruction quality for large values of $\lambda _L$, perhaps a poor combination with the choice of coefficient normalization.

### Alpha-matching

First- and second-order Taylor expansions were compared to assess $\alpha$-matching loss effectiveness. While higher-order terms improve pixel-space approximations, the gains are limited for large parameter values. This connects to Noether Prior and Type-II networks, underscoring the importance of correct inductive biases.

With $\alpha$-matching (cf. Eqn. 13), a first order Taylor expansion was used to estimate the pixel space transformation. This has the obvious drawback that the model is expected to perform better for small values of the sampled transformation parameter. If instead one adds a second-order term, extending the support of the matrix exponential around the identity, slight improvements in the output images are obtained. There was no improvement in the range of parameter values, however. What we do observe, is a shift in the correct generator for higher values of $\lambda _\alpha$ (Fig. 7). Not only does the correct generator appear for optimal $\lambda _\alpha$, but the orbit of reconstructed digits at test time is more robust.

### Complex transformations

Results on non-affine transformations, such as SCT (Fig. 5, bottom), demonstrate the model’s capability to handle complex symmetries. For composed transformations (e.g., rotation and scaling), the model detects multimodal distributions, capturing combined effects accurately: For compositions, we sampled from a categorical distribution for both rotation angle ($-45$ or 45 degrees) and scaling factor (0.5 or 1.5), resulting in a 4-modal distribution (Fig. 4e). Finally, we mention that the model is also capable of handling multiple-parameter groups in latent space, as illustrated in Fig. 2. It is possible to either include a closure factor by introducing an induced one-parameter group, i.e., $e^{t_{12} [G_1,G_2]}$, or omit it entirely. For these multi-parameter cases, only the first order Taylor term is implemented. The results for auxiliary reconstruction are very good, especially for SyMNIST (SuperSyMNIST reconstructions can be seen in Figure 3, bottom row), while splitting up the learnt generator into the expected one-parameter groups is not always guaranteed since other superpositions of correct generators are theoretically possible.

## Discussion

While correct generators were hard to obtain, the pixel space transformations match the augmentations very well, including the number of modes in the distribution of outputs from the *t*-network. From qualitative analysis of the results for translated samples, we conclude we recover the symmetry generator the best here, perhaps due to an observed slight bias towards keeping the drift terms non-zero. Rotations, scaling, shear, and SCTs are less conclusive, but recoverable for the right settings of the regularizers, in particular for high enough values and order of the alpha-matching term. In particular, small to medium transformation magnitudes, but not so small as to be below the aliasing threshold, one-parameter group augmentation transformed SyMNIST, with correctly tuned $\lambda _\alpha$ yields the correct symmetry generator in most cases. Reconstructions and transformation magnitude distributions are quantitatively very good, in particular the latter. Despite the MSE reconstruction loss having drawbacks, such as blurriness and misalignment with the theoretical objective, we observe relatively good results even in the SuperSyMNIST setting, suggesting good to excellent performance on the overall shape of an object, a promising result assuming one can disentangle texture and high frequencies from overall pose and, assumedly, low spatial frequencies. This is most likely rooted in the role of the reconstruction as an auxiliary task, the MSE loss having a smoothing effect. Additionally, we note that the model seems to learn “Platonic” digits, namely recognizable digits with different font or style than the expected one, that are transformed appropriately (this was not so clear in the GalaxSym dataset). Despite learning correct transformations in pixel space, the latent transformations are still hard to get exactly right for a single set of hyperparameters for multiple transformations, the latent transformations seem to still be underconstrained. From the $\alpha$-matching experiments, we deduce that this parameter is the most crucial to tune correctly, and we provide some quantitative results showcasing behavior beyond its optimal value. We expect that this term helps the coefficients flow towards a basin where the correct pixel-space transformations are reachable, although placing too much weight on this term might be counter-productive for large values of the parameters.

Finally, we note the choice of interpolation scheme and aliasing issues in latent space. Experimenting with different choices for interpolation, we do not observe major differences in the results shown above. Nevertheless, the codebase has options to change this in order to allow for further experimentation in this direction. We note that the latent patches do not seem to have learnt any geometric structure in latent space detectable by eye, despite the traversals in magnitude space being relatively stable and, in all cases, interpretable when decoded to pixel space.

## Supplementary Information

Below is the link to the electronic supplementary material.

Supplementary Information 1.

Supplementary Information 2.

Supplementary Information 3.

Supplementary Information 4.

## Data Availability

The dataset analysed is the widely available MNIST, which can be accessed through many libraries such as torchvision.datasets.MNIST. It can also be found at https://www.kaggle.com/datasets/hojjatk/mnist-dataset1. It contains 60,000 training images and 10,000 test images of handwritten digits, each in greyscale with a resolution of 28x28 pixels. The torchvision library was used to apply affine transformations to the MNIST images. For non-affine transformations, such as the SCT, the torch.nn.functional.grid_sample method was used.

## Appendix: Experimental details

For SyMNIST, the encoder and decoders were fully-connected MLPs with 512, 256, 128, 64 neurons in each hidden layer with LeakyReLU (0.2) activation functions. The output layer of the encoder and therefore the input layer of the decoder had size 81, for most of the experiments, or 25 for a couple of others (e.g., Fig. 4). The Adam optimizer^44^ was used with a learning rate of 0.001, batch size of 1024. All the results are shown for 1 channel in latent space, but more channels are possible and can improve the reconstruction quality. Wider and deeper MLPs, and various channels in latent space were all checked (1, 4, 16, and 64). 16 seemed to work best for MSE loss on the reconstructions, but there was no dramatic increase in predictive performance regarding the generators. LayerNorm was also used. All these options, including the ones discussed in the main text, can easily be toggled in the code accompanying this paper (https://github.com/alexgabel/LiePaper). For consistency, the same hyperparameters were used, namely $\lambda _Z=0.001, \lambda _R=1.0, \lambda _L=0.1, \lambda _\alpha = 0.001$, unless specified otherwise. For the GalaxSym experiments, we opted for identical architecture as well as a single channel, since this produced good reconstructions, and 81 latent pixels. The alpha-matching regularizer was 0.001 for all the results except for the shifted pairs, where a value of 1.0 yielded cleaner samples. The transformation magnitudes were uniformly sampled, with a maximum of 15 pixels, 90 degrees, and 1.5 scaling factor for translation, rotation, and scaling respectively. For the final rows (SuperGalaxSym) the alpha-matching regularizer was set to 1000.0.
