# Supplementary figures and images for: Type-II neural symmetry detection with Lie theory
Source: Sci Rep. 2025 Sep 29;15:33500. doi: 10.1038/s41598-025-17098-8 (PMC12480656; doi:10.1038/s41598-025-17098-8)

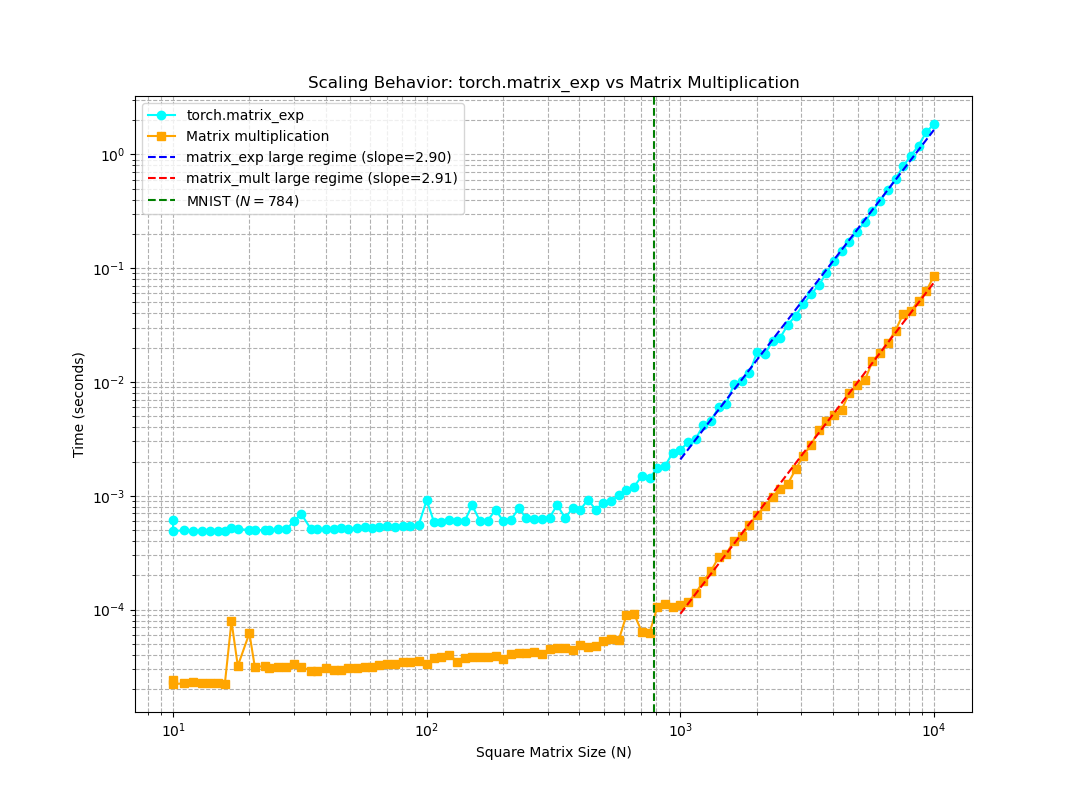

Supplement: Supplementary file 1 — Supplementary Information 1. [file 41598_2025_17098_MOESM1_ESM.png]

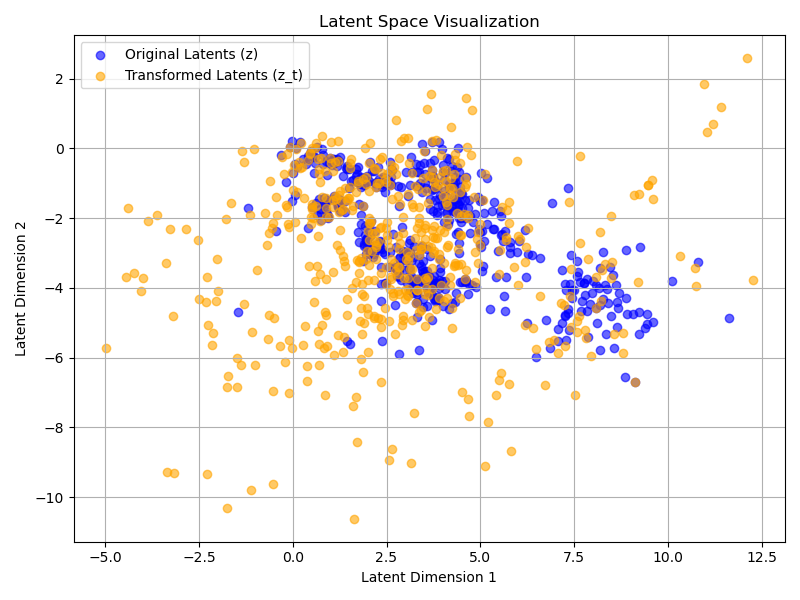

Supplement: Supplementary file 2 — Supplementary Information 2. [file 41598_2025_17098_MOESM2_ESM.png]

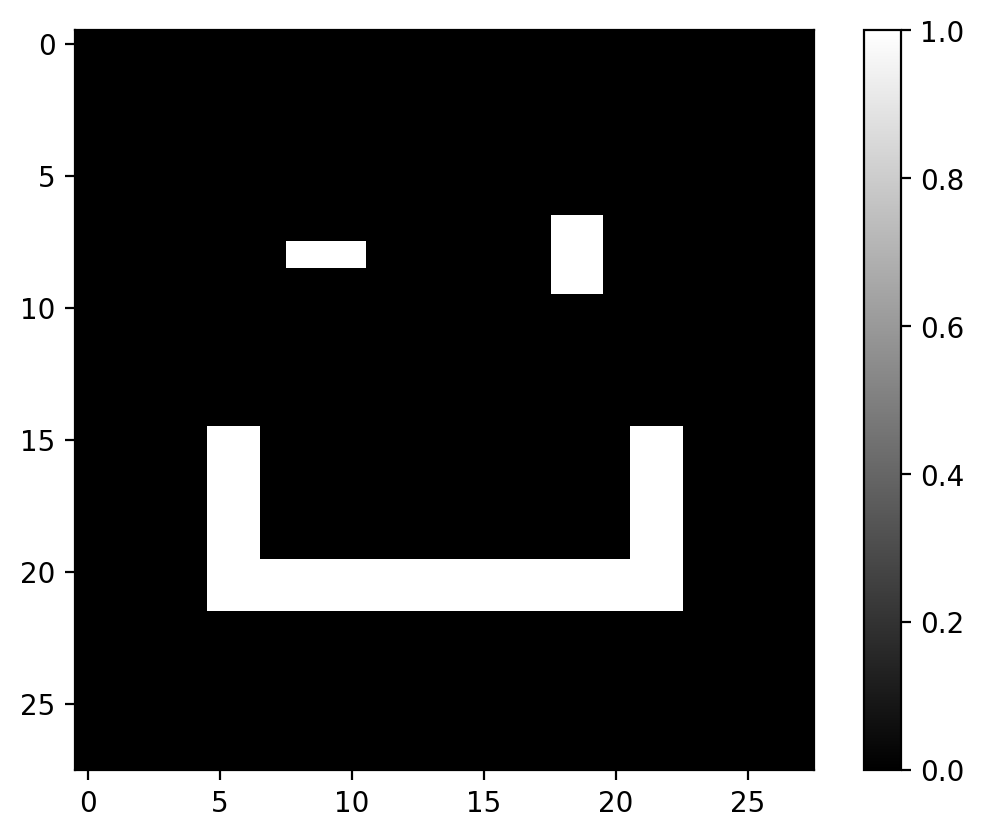

Supplement: Supplementary file 3 — Supplementary Information 3. [file 41598_2025_17098_MOESM3_ESM.png]

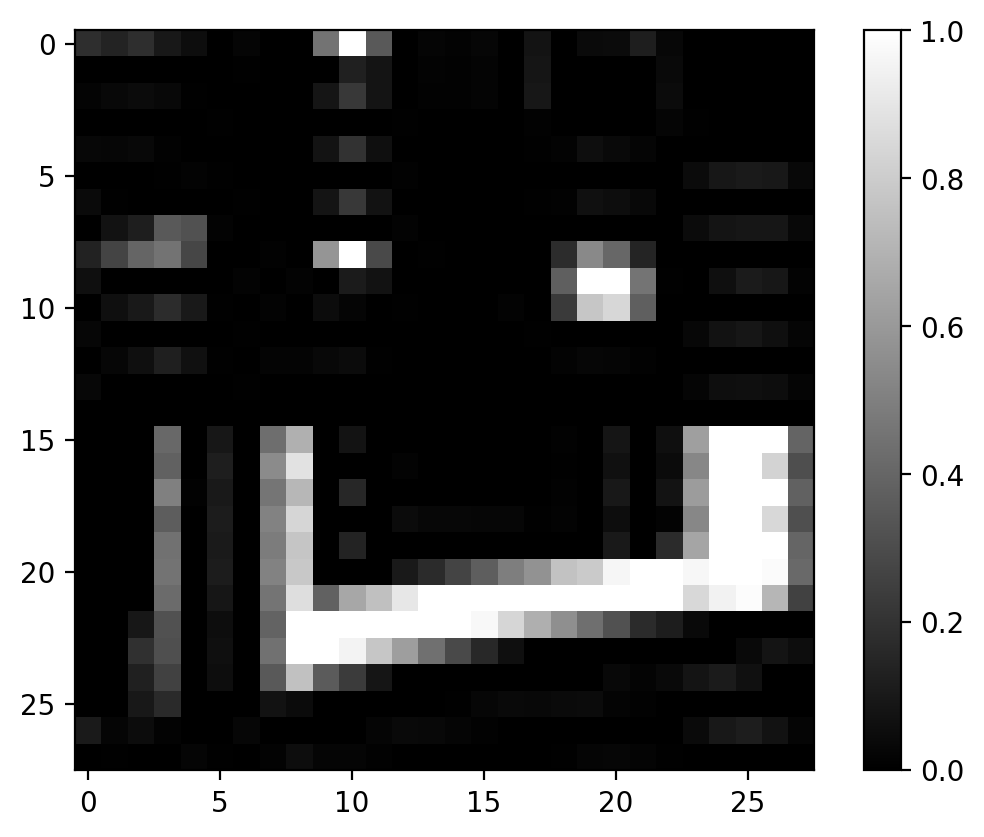

Supplement: Supplementary file 4 — Supplementary Information 4. [file 41598_2025_17098_MOESM4_ESM.png]
